# Expression of Concern: Case report: A rare case of tumor-to-tumor metastasis: metastatic lobular breast carcinoma to clear cell renal cell carcinoma

**DOI:** 10.3389/pore.2023.1611563

**Published:** 2023-11-09

**Authors:** Pathology & Oncology Research Editorial Office

Following publication, the authors contacted the Editorial Office stating that the findings reported in the article are no longer supported by the analyses. With this notice, Pathology and Oncology Research states its awareness of concerns regarding “**A rare case of tumor-to-tumor metastasis: metastatic lobular breast carcinoma to clear cell renal cell carcinoma**” published on 12 June 2023. This expression of concern has been posted while Pathology and Oncology Research awaits further clarification from the authors. It will be updated accordingly after that time.

